# Trauma-Associated Tinnitus: Audiological, Demographic and Clinical Characteristics

**DOI:** 10.1371/journal.pone.0045599

**Published:** 2012-09-26

**Authors:** Peter M. Kreuzer, Michael Landgrebe, Martin Schecklmann, Susanne Staudinger, Berthold Langguth, The TRI Database Study Group, Veronika Vielsmeier, Tobias Kleinjung, Astrid Lehner, Timm B. Poeppl, Ricardo Figueiredo, Andréia Azevedo, Ana Carolina Binetti, Ana Belén Elgoyhen, Marcelo Rates, Claudia Coelho, Sven Vanneste, Dirk de Ridder, Paul van de Heyning, Florian Zeman, Markus Mohr, Michael Koller

**Affiliations:** 1 Department of Psychiatry and Psychotherapy, University of Regensburg, Regensburg, Germany; 2 Department of Psychiatry, Psychosomatic Medicine and Psychotherapy, Sozialstiftung Bamberg, Bamberg, Germany; Emory Univ. School of Medicine, United States of America

## Abstract

**Background:**

Tinnitus can result from different etiologies. Frequently, patients report the development of tinnitus after traumatic injuries. However, to which extent this specific etiologic factor plays a role for the phenomenology of tinnitus is still incompletely understood. Additionally, it remains a matter of debate whether the etiology of tinnitus constitutes a relevant criterion for defining tinnitus subtypes.

**Objective:**

By investigating a worldwide sample of tinnitus patients derived from the Tinnitus Research Initiative (TRI) Database, we aimed to identify differences in demographic, clinical and audiological characteristics between tinnitus patients with and without preceding trauma.

**Materials:**

A total of 1,604 patients were investigated. Assessment included demographic data, tinnitus related clinical data, audiological data, the Tinnitus Handicap Inventory, the Tinnitus Questionnaire, the Beck Depression Inventory, various numeric tinnitus rating scales, and the World Health Organisation Quality of Life Scale (WHOQoL).

**Results:**

Our data clearly indicate differences between tinnitus patients with and without trauma at tinnitus onset. Patients suffering from trauma-associated tinnitus suffer from a higher mental burden than tinnitus patients presenting with phantom perceptions based on other or unknown etiologic factors. This is especially the case for patients with whiplash and head trauma. Patients with posttraumatic noise-related tinnitus experience more frequently hyperacousis, were younger, had longer tinnitus duration, and were more frequently of male gender.

**Conclusions:**

Trauma before tinnitus onset seems to represent a relevant criterion for subtypization of tinnitus. Patients with posttraumatic tinnitus may require specific diagnostic and therapeutic management. A more systematic and – at best - standardized assessment for hearing related sequelae of trauma is needed for a better understanding of the underlying pathophysiology and for developing more tailored treatment approaches as well.

## Introduction

Each year, approximately 1–2 million people experience a traumatic brain injury ( = TBI) in the United States [1], [2]. Apart from that, TBI is found among the most common war related injuries due to blast and concussion [3]. Recent studies revealed that 15.8% of service members in the Iraq war experienced traumatic brain injury [4], [5], in combat team samples an even higher prevalence ranging up to 22.8% was reported [6].

Trauma to the head – especially to the ear – is often associated with tinnitus [7], defined as an intermittent or constant sound sensation which cannot be attributed to an external sound source. In military personnel with TBI up to 38% reported comorbid tinnitus complaints [8]. A random sample of the Iraq war records revealed that 71% of soldiers experienced loud noises and that 15.6% had tinnitus [9], [10]. According to the American Tinnitus Association 3 to 4 million veterans suffer from tinnitus and compensation payments for combat related hearing loss [10] and tinnitus account for approximately US$ 1.2 billion per year [11]. But also in civil populations acoustic trauma is among the most frequently reported triggers for the development of chronic tinnitus [12], [13].

Notably, many different types of trauma can precede tinnitus onset. Noise trauma typically causes damage to the inner ear, and as a consequence leads to tinnitus [14]. Brain injuries can cause a variety of auditory symptoms such as hearing loss, tinnitus, and central deficits indicating the vulnerability of the auditory pathways to traumatic injury [15]. Moreover, also neck [16] injuries and emotional trauma [17], [18] are well known triggers of tinnitus [19], [20]. Therefore, we investigated the categories “noise trauma”, “whiplash”, and “head trauma”, and its combinations with regard to a large sample of tinnitus patients presenting in specialized tinnitus centers worldwide. The term “post-traumatic” or “trauma-associated” as mentioned in the present manuscript does not refer to the psychological aspects of trauma in the sense of “posttraumatic stress (disorder)”. It is rather applied to subsume the trauma categories “noise trauma”, “whiplash”, and “head trauma” (without further specification of its extent).

Tinnitus can vary in its phenomenological characteristics and in the amount of the related distress. It can cause severe distress on individuals and it has been shown to be correlated with sleeping disorders [21], depression [18], [22] and anxiety [23], [24], [25], [26], [27], [28], [29], [30]. It may affect the individual’s concentration and ability for attentional focusing and working memory [31], [32]. Development of tinnitus may even end up in suicidal attempts [33], [34], [35], [36]. Perceptual characteristics such as tinnitus loudness [37] or tinnitus frequency [38] only explain to a small extent the amount of tinnitus suffering. Other relevant factors include age at onset [39], personality factors [40] and coping behavior [41]. It has also been proposed that etiologic factors such as head and neck injuries related to tinnitus onset may exert an influence on the amount of tinnitus complaints [16].

In order to find out whether trauma and especially the different types of trauma at tinnitus onset are of relevance for perceptual, demographic and clinical characteristics of tinnitus a large worldwide sample of patients presenting at specialized tinnitus clinics has been investigated. The main objectives were to 1) to determine the percentage of cases of chronic tinnitus related to different types of trauma; 2) to describe the characteristics of these population and 3) to compare these characteristics with tinnitus patients whose tinnitus onset was not associated with trauma.

## Materials and Methods

The data presented in this study derive from the Tinnitus Research Initiative Database [42]. Data management was conducted according to the Data Handling Plan (TRI-DHP V07, May 9th, 2011). Data analysis was conducted according to the Standard Operating Procedure (TRI-SA V01, May 9^th^, 2011), thereby following a study-specific Statistical Analysis Plan (SAP-008, 20/12/2011) that was written according to the SAP template (TRI-SAP V01, May 9^th^, 2011). All documents can be accessed under http://database.tinnitusresearch.org/. Analysis details can found at the end of this section.

**Figure 1:**
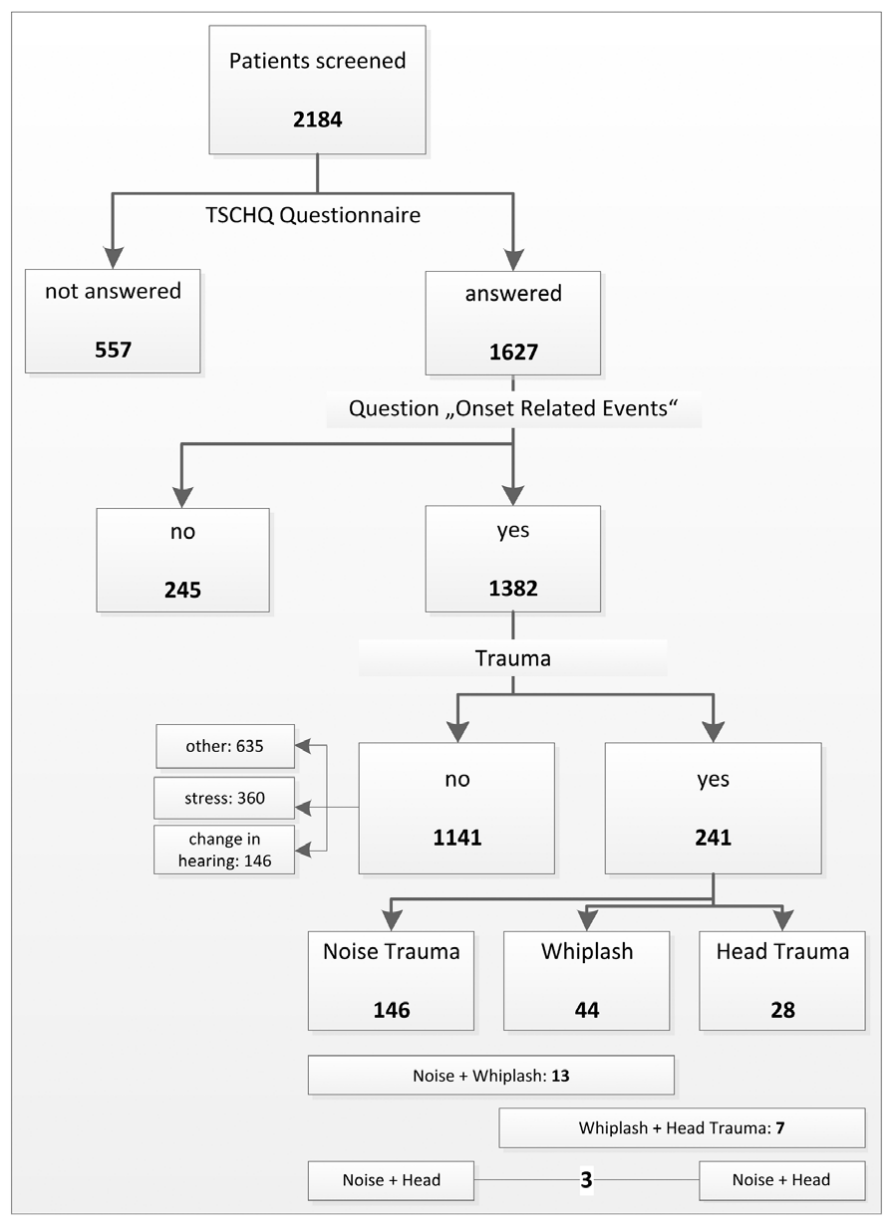
Patients’ flowchart, data provided in absolute numbers.

The default dataset import (November 1^st^, 2011) from the Tinnitus Research Initiative (TRI) Database consisted of 2,184 patients (see figure 1). Patients presented between 2005 and 2011 at different tinnitus centres worldwide (mainly from Brasil and Europe; center specific analysis could not be done due to irregular frequencies for the single trauma types with respect to centres; more detailed information about contributing tinnitus centres can be found at http://database.tinnitusresearch.org/en/map/map_en.php). Patients gave written informed consent to record their data in the database and to perform analyses with the data. The project has been approved by the local ethics committee of the Medical Faculty of the University of Regensburg. The database project is coordinated by the department of psychiatry and psychotherapy of the University of Regensburg, Germany, and the database server is located at the ManaTheam GmbH in Regensburg.

**Table 1:** Sample characteristics and clinical assessment scales (given as mean±standard deviation); significant results after Bonferroni correction for multiple testing are highlighted in bold letters.

	no trauma		noise	whiplash	head	other	F/χ ^2^	df	p
sample characteristics									
**age at presentation**	**54.3±13.8**		**49.3±15.1**	**54.4±8.1**	**47.6±14.6**	**52.9±12.8**	**4.589**	**4**	**.001**
**age at tinnitus onset**	**45.3±15.2**		**36.2±15.3**	**47.4±8.5**	**41.2±16.5**	**44.3±13.6**	**11.820**	**4**	**<.000**
**gender (female/male)**	**94/151**		**32/114**	**12/32**	**10/18**	**447/693**	**18.606**	**4**	**.001**
tinnitus and audiologic characteristics									
**duration in months**	**103.7±108.7**		**144.5±135.5**	**75.1±82.7**	**75.6±69.0**	**95.6±104.0**	**7.096**	**4**	**<.000**
preceding treatments	2.61±1.040		2.77±1.159	3.05±1.011	2.54±1.170	2.78±1.052	2.806	4	.042
pulsatile (no/yes with heartbeat/yes other than heartbeat)	196/22/19		115/11/18	32/4/6	22/3/1	899/123/106	5.861	8	.663
tone/noise/crickets/other	120/46/54/19		85/30/18/11	19/8/6/10	14/8/3/3	616/204/210/97	21.432	12	.044
maskable (no/yes)	55/161		22/104	5/30	7/13	244/734	6.750	4	.150
somatic modulation (no/yes)	169/71		97/48	19/23	15/12	751/377	11.626	4	.020
headache (no/yes)	155/81		82/60	21/22	8/17	686/426	14.544	4	.006
**vertigo/dizziness (no/yes)**	**179/57**		**86/53**	**19/23**	**11/15**	**732/369**	**25.953**	**4**	**<.000**
temporomandibular disorder (no/yes)	196/42		108/34	26/17	15/8	874/237	13.173	4	.010
**neck pain (no/yes)**	**123/119**		**61/82**	**5/38**	**10/17**	**482/630**	**23.573**	**4**	**<.000**
**other pain (no/yes)**	**169/70**		**87/57**	**19/22**	**12/16**	**632/465**	**20.258**	**4**	**<.000**
**current psychiatric treatment (no/yes)**	**226/17**		**125/20**	**35/8**	**17/10**	**945/180**	**24.156**	**4**	**<.000**
**sound tolerance (low/high)**	**178/63**		**73/73**	**28/15**	**16/11**	**732/398**	**23.025**	**4**	**<.000**
**painful sounds (no/yes)**	**133/85**		**49/87**	**11/23**	**9/18**	**441/569**	**31.384**	**4**	**<.000**
hearing level (median HL)	23.2±20.31		21.4±13.38	18.8±11.83	18.7±9.92	20.7±13.51	1.395	4	.233
tinnitus questionnaires									
**TQ**	**36.2±18.53**	**42.5±18.14**		**49.2±14.74**	**51.8±20.81**	**40.6±17.37**	**7.090**	**4**	**<.000**
**THI**	**42.8±24.82**	**51.5±23.44**		**55.3±23.70**	**60.7±24.44**	**48.0±22.55**	**6.770**	**4**	**<.000**
**BDI**	**9.8±8.72**	**11.6±8.07**		**15.4±10.20**	**15.6±9.19**	**11.1±8.56**	**5.733**	**4**	**<.000**
**WHOQOL domain 1**	**14.7±3.27**	**13.9±3.22**		**12.7±3.51**	**12.5±3.63**	**14.4±2.98**	**4.735**	**4**	**.001**
WHOQOL domain 2	14.2±2.98	13.9±2.52		12.6±2.66	12.6±2.78	14.0±2.74	3.154	4	.014
WHOQOL domain 3	15.0±3.22	13.9±3.03		13.3±3.27	13.5±2.38	14.5±3.17	3.094	4	.015
**WHOQOL domain 4**	**15.7±2.80**	**15.0±2.56**		**14.7±2.90**	**13.5±3.06**	**15.8±2.37**	**6.487**	**4**	**<.000**
numeric rating scales									
loudness	6.2±2.34	6.4±2.34		6.9±2.16	6.9±2.29	6.4±2.13	1.472	4	.208
discomfort	6.9±2.55	7.0±2.29		7.7±1.94	7.4±2.15	7.0±2.26	1.359	4	.246
annoyance	6.4±2.62	6.5±2.36		7.4±2.05	7.3±2.23	6.6±2.34	2.009	4	.091
ignorability	6.6±2.90	6.6±2.71		7.7±2.03	7.2±2.87	6.7±2.57	1.774	4	.131
unpleasantness	6.5±2.61	6.6±2.45		7.5±1.81	7.2±2.29	6.6±2.37	2.046	4	.086

The variable “onset related event” was deduced from the question of the Tinnitus Sample Case History Questionnaire (TSCHQ; 45) “Was the initial onset of your tinnitus related to:” with the pre-formulated answers “loud blast of sound”, “whiplash”, “change in hearing”, “stress”, “head trauma”, and “other”. Patients who did not answer the TSCHQ were excluded from analysis resulting in a sample of 1,604 patients (see figure 1). Patients were grouped according to their self-reported onset related events (see figure 1).

We built the five categories “no trauma”, “noise trauma”, “whiplash”, and “head trauma”, and “other tinnitus related onset”. If one patient reported multiple events including one single trauma this patient was subsumed to the group of this trauma. Patients with multiple traumata were excluded from primary analysis (n = 23; 1.4%) (see figure 1).

Assessment was performed before the first consultation in the tinnitus clinics and included the Tinnitus Sample Case History Questionnaire (TSCHQ), the Tinnitus Handicap Inventory (THI) [43], the Tinnitus Questionnaire (TQ) [44], the Beck Depression Inventory (BDI) [45], and four domains of the World Health Organisation Quality of Life Scale (WHOQoL). The different domains of the WHOQOL measure 1^st^ “physical health”, in particular activities of daily living, dependence on medicinal substances and medical aids, energy and fatigue, mobility, pain and discomfort, sleep and rest, work capacity; 2^nd^ “psychological health”, in particular bodily image and appearance, negative/positive feelings, self-esteem, spirituality/religion/personal beliefs, thinking, learning, memory and concentration; 3^rd^ “social relationships”, in particular personal relationships, social support, sexual activity; and 4^th^ “environmental factors”, in particular financial resources, freedom, physical safety and security, health and social care, home environment, opportunities for acquiring new information and skills, opportunities for recreation, physical environment, and transport [46].

Subjectively perceived loudness, discomfort, annoyance, ignorability, and unpleasantness were assessed by numeric rating scales (range: 0–10).

In addition to these variables, we were interested in the demographic characteristics age, gender and age at tinnitus onset. Furthermore, we investigated the tinnitus duration until presentation at our clinic, the way of onset (gradual/abrupt), number of preceding treatments until presentation, tinnitus characteristics such as pulsatile or non-pulsatile sound perception, tonal or noise-like tinnitus, the possibility of masking the tinnitus by music or sounds and the ability of modulating the tinnitus by somatic manoeuvres. Comorbid symptoms of tinnitus such as headaches, vertigo/dizziness, temporomandibular disorders, neck pain or other pain symptoms were asked for. Hyperacousis was checked by the following TSCHQ-items (number 28 and 29): a) “Do you have a problem tolerating sounds because they often seem much too loud? That is, do you often find too loud or hurtful sounds which other people around you find quite comfortable?” with the predetermined response possibilities “never/rarely/sometimes/usually/always” and b) “Do sounds cause you pain or physical discomfort? “yes/no/I don’t know”.

In addition, current psychiatric treatment was assessed. The mean hearing level (dB hearing level (pure tone audiogram) over eight frequencies (0.125/0.250/0.500/1/2/4/6/8 kHz) of both ears was documented and averaged.”

**Figure 2:**
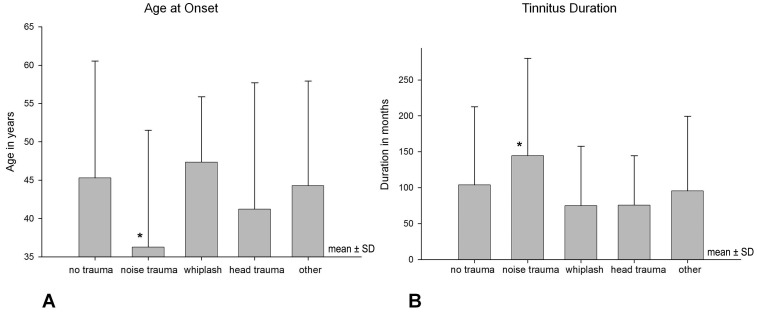
Age at Tinnitus Onset and Tinnitus Duration (mean ± SD). **A** * Statistically significant between-groups contrasts (p<0.001) to groups “no trauma”, “whiplash”, and “other”. **B** * Statistically significant between-groups contrasts (p<0.005) to groups “no trauma”, “whiplash”, “head trauma”, and “other”.

**Figure 3:**
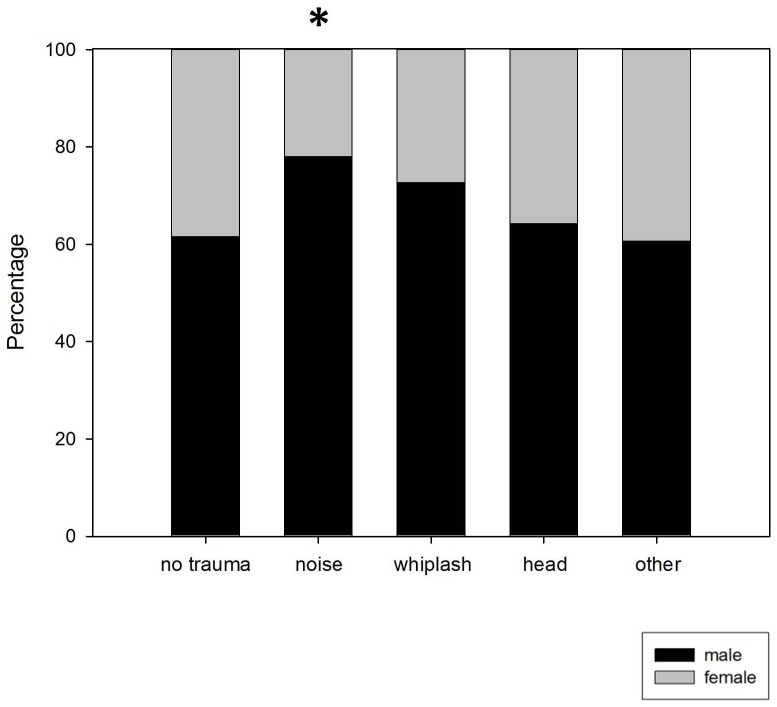
Gender distribution shown in percentage of male/female patients. * Proportion of male patients higher than statistically expected.

**Figure 4:**
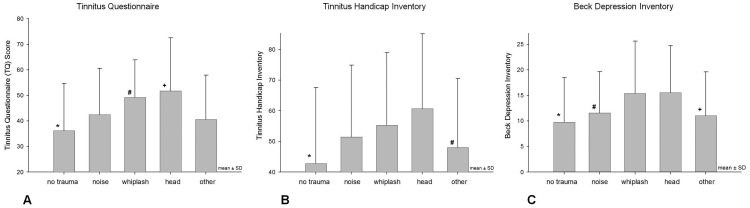
Clinical Questionnaire Assessment Scales at Presentation (mean ± SD). **A** * Statistically significant between-groups contrasts to all other groups (p<0.005). ^#^ Statistically significant between-groups contrasts to groups “no trauma”, “noise”, and “other”(p<0.05). ^+^ Statistically significant between-groups contrasts to groups “no trauma” and “other” (p<0.05). **B** * Statistically significant between-groups contrasts to all other groups (p<0.005). ^#^ Statistically significant between-groups contrasts to groups “no trauma”, “whiplash”, and “head” (p<0.05). **C** * Statistically significant between-groups contrasts to all other groups (p<0.05). ^#^ Statistically significant between-groups contrasts to groups “no trauma”, “whiplash”, and “head” (p<0.05). ^+^ Statistically significant between-groups contrasts to groups “no trauma”, “whiplash”, and “head” (p<0.05).

**Figure 5:**
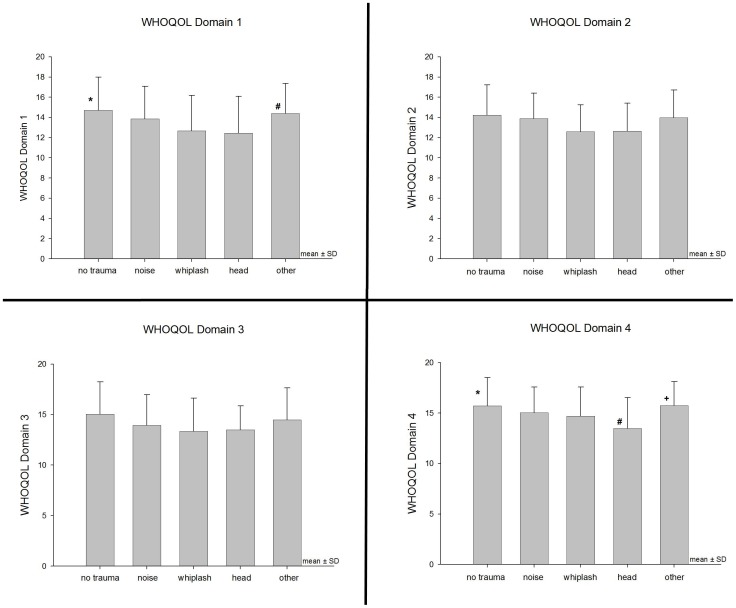
WHOQOL Questionnaire. Domain 1 * Statistically significant between-groups contrasts(p<0.05) to groups “noise”, “whiplash”, and “head”. ^#^ Statistically significant between-groups contrasts (p<0.05) to groups “whiplash” and “head”. **Domain 4** * Statistically significant between-groups contrasts (p<0.05) to groups “noise” and “head”. ^#^ Statistically significant between-groups contrasts (p<0.05) to groups “noise”, “whiplash” and “head”. ^+^ Statistically significant between-groups contrasts (p<0.05) to group “noise”.

If no data were available at the screening visit (first consultation), we used data from the baseline visit of a clinical intervention. If both screening and baseline data were available we used the mean of both time points. For statistical analyses we used analyses of variance (ANOVAs) for continuous variables (e.g., age) and chi-square-tests for categorical variables (e.g., gender) with the variable onset (no trauma/noise/whiplash/head/other) as group variable (table 1). Significance threshold was set to a Bonferroni corrected level of 0.0016 (0.0016 = 0.05/31 included variables). In case of significant results in the ANOVA, post-hoc tests were performed by using t-tests for continuous variables and using z-standardized residuals of the frequencies of the single cells of the chi-square-tests for categorical variables. These post-hoc tests are displayed graphically (figure 2 to 5). For post-hoc tests significance threshold was set to an uncorrected level of 0.05.

## Results

A total of 1,604 patients answered the TSCHQ-Question concerning onset-related events thus providing a self-report of possible triggering factors of the phantom perception. 241 individuals reported a trauma-associated tinnitus onset. 146 reported a history of noise trauma, 44 a history of whiplash, and 28 a history of head trauma as an *isolated* trigger for their tinnitus. Detailed information is given in figure 1.

We found significant group effects for the variables age at presentation, age at onset, gender, tinnitus duration, vertigo/dizziness, neck pain, other pain symptoms, current psychiatric treatment, TQ, THI, BDI, WHOQOL domain 1 and 4, and both hyperacousis-questions regarding “sound toleration” and “sounds causing pain and physical discomfort”.

Groups did not differ for the variables “number of preceding treatments”, “pulsatile vs. non-pulsatile tinnitus character”, “tonal/noise-like/cricket-like tinnitus”, “maskability”, “somatic modulation”, “headache”, “temporomandibular joint disorders (TMJD)”, “hearing level”, WHOQOL domains 2 and 3, and the five numeric tinnitus rating scales regarding the aspects “loudness”, “discomfort”, “annoyance”, “ignorability”, and “unpleasantness”.

As patients with multiple trauma history (n = 23; see figure 1) have been excluded from analysis as described in the methods section of this manuscript, we additionally calculated thirty (as shown in table 1) group contrasts of single trauma cases vs. multiple trauma cases (n = 218; subsuming all patients with noise or head trauma or whiplash) resulting in no significant results (lowest p-values = .017 and.028 (uncorrected for multiple comparisons) for the items “preceding treatment approaches” and tinnitus questionnaire total score, respectively.

Comprehensive information about clinical characteristics, questionnaire ratings and audiological data is given in table 1.

## Discussion

### Occurrence of Tinnitus Onset Related Traumata

With a percentage of 4.9% ( = (44+28+7)/1604; see figure 1) patients suffering from a tinnitus induced by whiplash or head trauma (or a combination) represent only a small subsample in our database derived from several tinnitus centers worldwide participating in the Tinnitus Research Initiative (TRI). In an earlier study, Folmer and Griest reported a prevalence of a history of head and neck trauma in more than 10 percent of the patients presenting in the specialized tinnitus clinic in Portland, Oregon [16]. Vice versa, in the literature there is evidence that tinnitus is found to be highly prevalent in patients with a history of trauma. Flint *et al.* investigated the incidence of persisting auditory and vestibular sequelae in a group of 30 young adults (aged 21–45 years) recovering from traumatic brain injury, that had taken place previously (range 19 months to 27 years). A variety of sequelae to TBI were reported including tinnitus (53%), vestibular dysfunction (83%), abnormal facial sensory symptoms (27%) and intolerance to loud/sudden noises (87%) [19]. Ten (33%) participants demonstrated significant sensorineural hearing impairment in addition to speech recognition performance significantly worse than would have been predicted from their hearing impairment [19].

But also in total, given the high prevalence of head, neck and noise trauma and the frequent occurrence of tinnitus after these traumas the rate of reported trauma at tinnitus onset in our sample is very low. Different explanations may account for this finding: a) trauma at tinnitus onset may be underreported by patients especially when there was a delay between trauma and tinnitus onset; b) patients with posttraumatic tinnitus may be preferentially encountered in specific settings such as military medicine or occupational medicine; c) posttraumatic tinnitus might be underestimated by treating physicians with respect to other trauma-related symptoms and therefore these patients might not be referred to tinnitus clinics and be underrepresented in this study population; d) Patients with trauma-associated tinnitus experience tinnitus frequently but rarely present themselves to specialized tinnitus centres because they do not suffer a lot from it compared to other trauma-related impairments; The last explanation is rather unlikely as in our study sample patients with trauma-associated tinnitus showed greater symptom-related burden (see figure 4).

We are aware that our study can only provide a vague estimation of the prevalence of trauma preceding tinnitus onset. First, our analyses rely entirely on patients’ self reports; secondly even if our sample is large and derives from many centers world-wide it is biased since it reflects the patient population in specialized tinnitus clinics. Therefore, further prospective and population based studies are needed for a more precise estimation of the prevalence of trauma before tinnitus onset.

### Summary of the TRI Database Findings

Posttraumatic tinnitus was associated with higher distress levels reflected by higher scores in TQ, THI, BDI, and WHOQoL subscores 1 and 4 as compared to tinnitus patients without trauma at tinnitus onset. These findings reached statistical significance for whiplash and head trauma (TQ, THI, BDI, WHOQOL Dom1 and Dom4) (see figures 4 and 5). This is in line with the findings of the study of Folmer and Griest, who reported both higher Tinnitus Severity Index Scores. However, these authors found also more pronounced tinnitus loudness (measured by 1-to-10 scales and tinnitus matching) in patients suffering from trauma-associated tinnitus [16]. Whereas, our data did not show any effects in tinnitus ratings as elicited by numeric rating scales. In addition, these patients reported greater difficulties with concentration, memory, and clear thinking [16]. Possibly, trauma-associated tinnitus might constitute a subtype connected with higher symptom-related distress levels. Another explanation would be that the higher impairment of posttraumatic tinnitus is not directly associated with a specific trauma associated pathophysiology but rather reflects dysfunctional coping strategies. A “fateful” tinnitus onset without any triggering factors might be tolerable, habituable and integrable more easily than a trauma-associated onset which is often accompanied by strong emotions such as surprise, helplessness, pain, and fear, especially when the trauma has not been caused by the patient but by a second party. In many of these cases, patients suffer not only from a bodily damage but tend to regard themselves as “victims” and spend much time with ruminating thoughts about the traumatic event and its preventability such as “If I hadn’t gone there and wouldn’t have done that… I would not have met this…“. Similarly, the expectation for compensation payments may influence habituation. These dysfunctional coping strategies may particularly explain the elevated depression scores in the BDI after head and whiplash trauma. The participation rate in psychiatric treatment reflects this situation, indicating that trauma patients in our sample underwent current psychiatric treatment more frequently than patients without trauma-related onset (see table 1). However, this finding might be biased by psychiatric trauma consequences unrelated to tinnitus. Also, the WHOQOL Quality of Life Questionnaires showed similar patterns like the THI, TQ, and BDI. Apparently, trauma patients as a whole suffered from a more pronounced impairment of their quality of life than tinnitus patients of other etiologies. This pattern was similar for all of the four WHOQOL domains (see Figure 5).

**Figure 6:**
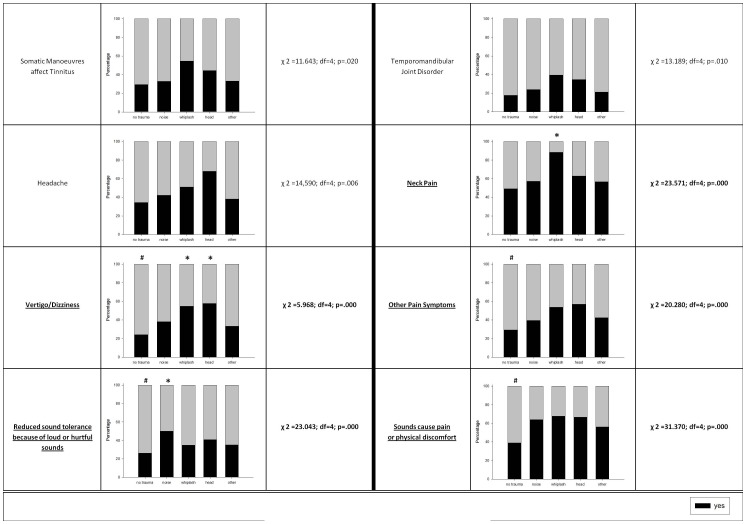
Percentage of accompanying factors of tinnitus with respect to different trauma-associated and non-trauma-associated tinnitus patients' groups. Significant results (after Bonferroni correction for multiple testing) are highlighted in bold and underlined letters; * higher proportion of patients with yes-answer than expected; ^#^ lower proportion of patients with yes-answer than expected.

Patients without tinnitus related onset events (including trauma and non-trauma events) reported less frequently that sounds can cause them pain or physical discomfort (see figure 6). Furthermore, the tolerance to loud or hurtful sounds is reduced especially in the subgroup of noise trauma patients (see figure 6). As patient groups did not differ in their hearing levels, recruitment phenomena are considered unlikely to account for these symptoms. In particular, there was no significant between-groups contrast of hearing loss levels at 4 kHz (F = 0.906; df = 4; p = 0.460) and no at the remaining frequencies either (F = 1.414; df = 4; p = 0.227) suggesting that no typical c5-dip [47] was present in noise trauma patients. Rather, the impairment assessed by both questionnaire items suggests a more frequent occurrence of hyperacousis in patients with noise related posttraumatic tinnitus, thus indicating differences in central auditory processing [48]. In this regard our findings of an increased rate of hyperacousis in posttraumatic tinnitus correspond well with earlier studies demonstrating high rates of intolerance to loud sounds after TBI [19]. These findings indicate that in TBI in addition to potential cochlear damage the direct impact on the central auditory pathways is relevant for the etiology of posttraumatic tinnitus. As also mentioned above, no difference in cumulative hearing loss between patients with trauma and non-trauma-groups was observed in our study (see table 1). On a descriptive level, patients without a history of trauma showed higher levels of cumulative hearing loss than patients affected by noise trauma but this difference did not reach significance. This is surprising because it has been reported that acoustic trauma and blast injuries result typically in high-frequency hearing loss [15], [47]. However, Nicolas-Puel *et al.* reported similar findings in a sample of 555 patients attending the specialized Tinnitus Clinic in Montpellier, France. Patients with a reported history of noise trauma (17% of the total sample) showed symmetrical hearing loss without differences in lateralization of the tinnitus percept. As in our study, this subset of patients was mainly male and on average 10 years younger than other tinnitus patients. Possibly, young men tend to live an active life and execute risky behavior more frequently thus undergoing an increased risk for accidents. In this French study hearing loss of patients with noise trauma was significantly less pronounced than that measured in the other patients [49]. The observed less pronounced hearing loss in tinnitus patients with noise trauma may be a hint for the relevance of the temporal dynamics of hearing loss for tinnitus generation. As compared with slowly progressing hearing loss in presbyacousis, noise trauma causes a sudden hearing impairment which might trigger specific neuroplastic mechanisms in the brain which in turn induce tinnitus. The induced tinnitus may then persist, even if hearing function recovers in many patients.

Further studies in humans and experimental studies in animals demonstrate that the extent of permanent threshold shift after trauma is variable and may depend on the type of trauma. Nölle *et al.* reported in a sample of 31 patients (24–56 years) after a history of blunt head trauma that initial sensorineural hearing loss (as a result of the inner ear fluid concussion) was transiently perceived only. Auditory brainstem responses (ABR) were normal in all patients, but 76% experienced lowered loudness discomfort levels. The authors stated that blunt trauma of the head can lead to auditory dysfunction, probably as a result of diffuse axonal injury of the central auditory pathway [50]. In contrast, Nageris *et al.* reported in a sample of 73 patients exposed to physical trauma in case of explosion, that 78% experienced high-frequency accentuated sensorineural hearing loss with only 7% improving over time [51]. Apparently, the correlation of head and acoustic trauma with hearing loss strongly depends on the specific form of trauma and its *extent,* so a more detailed, structured and - at best - standardized assessment of trauma-related information is considered vital for the drawing of outlines both in post-trauma research and the definition of therapeutic needs and the prediction of clinical outcomes.

In view of the findings described above patients with noise trauma are more frequently of male gender. Those with a history of noise trauma are younger and describe an earlier onset and longer tinnitus duration at first consultation in a contributing tinnitus center of the TRI consortium. This is well in line with the findings of Nicolas-Puel *et al.,* from a sample presenting at the tinnitus clinic in Montpellier. The patients presenting with noise-induced tinnitus were reported to be mainly male and on average were 10 years younger than other tinnitus patients. In contrast to our results, these patients were reported to differ also in their perceptional characteristics mainly suffering from bilateral high-pitched “whistling” tinnitus in correlation with their high-frequency hearing loss [52]. Especially in young people relevant noise exposure from recreational activities is expected. Between 50 and 70% of young people who expose themselves to loud recreational noise have temporarily experienced tinnitus [53]. Up to 75% of Disc jockeys develop tinnitus, They develop hearing loss both at high frequencies and at low frequencies and have tinnitus of the same sound spectra [54].

A further clinical observation was the fact that patients suffering from trauma-associated tinnitus reported pain symptoms more frequently than other non-trauma-patients. Whiplash patients indicated coincidence of neck pain at a significantly higher rate than all other groups (see figure 6). Vertigo and dizziness were more prevalent in patients suffering from tinnitus associated with whiplash and head trauma as already reported in the literature [55]. These findings support the notion of a generally strong correlation of tinnitus and pain syndromes [56] which might be even more pronounced in patients with trauma-associated tinnitus and merits careful prospective observational studies in the future.

### Conclusion and Outlook

Our data clearly indicate differences between tinnitus patients with and without trauma at tinnitus onset. Patients suffering from trauma-associated tinnitus suffer from a higher mental burden than tinnitus patients presenting with phantom perceptions based on other or unknown etiologic factors. This is especially the case for patients with whiplash and head trauma. Patients with posttraumatic noise-related tinnitus experience more frequently hyperacousis, were younger, had longer tinnitus duration, and more frequently male.

Thus, trauma before tinnitus onset seems to represent a relevant criterion for subtypization of tinnitus. Patients with posttraumatic tinnitus may require specific diagnostic and therapeutic management. A more systematic and – at best - standardized assessment for hearing related sequelae of trauma is needed for a better understanding of the underlying pathophysiology and for developing more tailored treatment approaches as well.
